# Exploring the Role of Hormones and Cytokines in Osteoporosis Development

**DOI:** 10.3390/biomedicines12081830

**Published:** 2024-08-12

**Authors:** Egemen Umur, Safiye Betül Bulut, Pelin Yiğit, Emirhan Bayrak, Yaren Arkan, Fahriye Arslan, Engin Baysoy, Gizem Kaleli-Can, Bugra Ayan

**Affiliations:** 1 Department of Biomedical Engineering, İzmir Democracy University, İzmir 35140, Türkiye; 2 Department of Biomedical Engineering, Bahçeşehir University, İstanbul 34353, Türkiye; 3 Department of Cardiothoracic Surgery, Stanford University, Stanford, CA 94305, USA

**Keywords:** osteoporosis, cytokines, hormones

## Abstract

The disease of osteoporosis is characterized by impaired bone structure and an increased risk of fractures. There is a significant impact of cytokines and hormones on bone homeostasis and the diagnosis of osteoporosis. As defined by the World Health Organization (WHO), osteoporosis is defined as having a bone mineral density (BMD) that is 2.5 standard deviations (SD) or more below the average for young and healthy women (T score < −2.5 SD). Cytokines and hormones, particularly in the remodeling of bone between osteoclasts and osteoblasts, control the differentiation and activation of bone cells through cytokine networks and signaling pathways like the nuclear factor kappa-B ligand (RANKL)/the receptor of RANKL (RANK)/osteoprotegerin (OPG) axis, while estrogen, parathyroid hormones, testosterone, and calcitonin influence bone density and play significant roles in the treatment of osteoporosis. This review aims to examine the roles of cytokines and hormones in the pathophysiology of osteoporosis, evaluating current diagnostic methods, and highlighting new technologies that could help for early detection and treatment of osteoporosis.

## 1. Introduction

Osteoporosis is a common bone disease for both men and women that occurs due to impaired bone structure or tissue and increases the risk of fractures [1]. Cytokines, hormones, and growth factors affect bone homeostasis, and play direct and indirect roles in osteoporosis diagnosis [2]. According to the WHO, osteoporosis is diagnosed when BMD is equal to 2.5 SD or lower than the average value for young and healthy women (T score of <−2.5 SD) [3]. A recent study indicates that the global prevalence of osteoporosis and osteopenia is 19.7% [4] and the economic burden of osteoporosis worldwide was projected to cost 56.9 billion USD in 2019 according to an economic model [5]. Therefore, effective diagnostic methods are urgently required. The role of cytokines and hormones in bone density has raised interest in osteoporosis diagnosis; however, since their upregulation and downregulation has been implicated in many diseases, including inflammation, cancer, dermatitis, pulmonary embolism, and menopause, detection of these biomarkers is not proper and selective as a diagnostic tool [6,7,8,9]. 

Cytokines regulate immune and inflammatory responses within the body through intricate networks [10]. In osteoporosis, cytokines, among osteoclasts and osteoblasts, including Tumor Necrosis Factor (TNF), the interleukin (IL) family, and growth factors (GFs), play crucial roles in bone remodeling, with the RANKL/RANK/OPG axis being essential within the cytokine networks and signaling pathways [11]. The equilibrium within RANKL/RANK/OPG controls the differentiation and activation of osteoclasts [12]. For instance, Ginaldi et al. revealed that IL-33 levels in postmenopausal women were significantly lower than in healthy controls (3.53  ±  2.45 vs. 13.72  ±  5.39 pg/mL), and they suggest that IL-33 is crucial for maintaining bone health [13]. Additionally, as a hormone secreted by osteoclasts and osteoblasts, fibroblast growth factors (FGF-23) can provide new treatment options for osteoporosis [14]. Hormones such as estrogen, parathyroid, testosterone, and calcitonin hormones significantly influence bone density. Estrogen deficiency is known for reducing bone resorption and inhibiting the development of new basic multicellular units (BMUs) [15]. The parathyroid hormone (PTH) activates bone resorption by osteoclasts and bone formation by osteoblasts and acts as a therapeutic agent [16]. Although androgens are considered the primary sex hormones in men, they block IL-6 cytokines and encourage osteoblast proliferation along with differentiation with studies showing that testosterone deficiency is associated with a risk of osteoporosis [17,18]. Finally, the calcitonin hormone reduces the reabsorption of calcium (Ca) and phosphate through kidneys, inhibiting osteoclast activity and bone resorption [19]. These findings regarding the roles of cytokines and hormones indicate that targeting these molecules in the diagnosis and therapy of osteoporosis is essential.

This review focuses on the development of osteoporosis and the role of cytokines and hormones in the pathophysiology of osteoporosis. By elucidating their interactions in bone resorption and remodeling, and their effect on osteoclasts and osteoblasts, we aim to provide new insights on studies concerning this particular disease. Furthermore, a deeper understanding of the mechanisms involved in osteoporosis will be significant in future research including early diagnosis and therapeutics, ultimately improving patient outcomes.

## 2. Pathomechanism of Osteoporosis

Bones have a wide variety of functions, including supporting the body structure and protecting vital organs [20]. They are involved in the storage and release of chemical elements and the production of red and white blood cells through hematopoiesis in the bone marrow [21]. Bones also act as a reservoir for minerals, especially Ca, and serve as a framework for muscles, tendons, ligaments, and joints to generate and transfer the force necessary for movement [22]. Additionally, bones play a crucial role in hearing through the ossicles located in the middle ear [23].

Bone tissue consists of four cell types: osteoblasts, osteocytes, osteoclasts, and bone lining cells. These cells are activated by various signaling molecules such as the receptor activator of RANKL and macrophage colony-stimulating factor (M-CSF). Osteoblasts synthesize and secrete bone matrices. Osteoblast activities are regulated by and stage-specific to hormones including PTH [24,25], vitamin D3 [26], estrogen [27,28], and glucocorticoids [29,30]. Bone tissue also consists of a calcified extracellular matrix (ECM) and organic and inorganic matters. While collagen type I, proteoglycans, and glycoproteins (such as osteonectin and osteocalcin) form the organic part, Ca, hydroxyapatite, bicarbonate, citrate, magnesium, potassium, and sodium ions form the inorganic part. The inorganic components, particularly Ca and phosphate, contribute to the mineralized matrix that provides the hardness and durability of bone [31]. 

The structural integrity and strength required for bone function are formed by the mineralized matrix consisting of hydroxyapatite crystals, consisting of the combination of calcium phosphate and calcium carbonate and settled between collagen fibers. Collagen fibers and proteins, the organic components that comprise bone, are synthesized by osteoblasts. Collagen fibers provide bone flexibility and durability by serving as a structural framework within the mineralized matrix [32]. Proteins such as osteocalcin, osteonectin and osteopontin play crucial roles in bone formation, integrity, mineralization, remodeling, and maintaining bone mass. Studies have shown that osteocalcin levels indicate bone formation rates and are directly proportional to BMD [33]. Since osteocalcin levels are affected during treatments such as alendronate, it can be used as a biomarker for postmenopausal osteoporosis treatment [34]. Osteonectin, also known as secreted acidic and cysteine-rich protein (SPARC), acts as a crystal nucleator and serves as a controller of cellular activities, playing roles in tissue remodeling, repair, development, and cell turnover [35]. High osteopontin levels are associated with low BMD in postmenopausal women, affecting bone health. Studies indicate that osteopontin acts as a signal transducer in regulating bone metabolism and diseases like osteoarthritis and osteoporosis [36]. Clinical studies have shown that osteopontin can be used as a biomarker for the early diagnosis of osteoporosis, especially in postmenopausal women [37].

**Figure 1:**
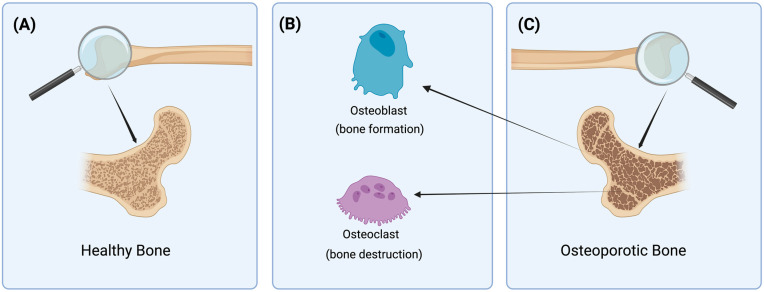
Schematic representation of healthy bone (**A**), the two main cell types involved in bone remodeling: osteoblasts and osteoclasts (**B**) and osteoporotic bone (**C**). A healthy bone is characterized by a well-defined, thick trabecular structure, sufficient mineralization, and minimal signs of fractures or degradation. The structure of osteoporotic bone is characterized by advanced bone loss and weakening, as well as significant reductions in density and thickness of trabecular bone, which results in the appearance of porous and fragile bone. This figure was created using https://app.biorender.com/, accessed on 18 June 2024.

The bone cycle describes the continuous process of bone resorption, migration, and formation, which ensures the renewal and maintenance of skeletal health (Figure 1A). Osteoclasts are responsible for the breakdown of old or damaged bone tissue, while osteoblasts enable bone formation by secreting a new bone matrix to replace the resorbed bone [38]. Osteocytes, as mature bone cells, regulate the activity of osteoblasts and osteoclasts and maintain bone health [39]. Bone lining cells play a role in clearing Howship lacunae and initiating bone formation, contributing to the overall bone restructuring process [40]. They also contribute to the balanced continuity of the bone cycle by modulating the expression of genes and proteins involved in bone remodeling, such as RANKL and OPG [41]. 

Osteocytes are the most abundant cellular components in bones and constitute more than 95% of bone tissue. They function as mechanosensory cells in bone and regulate bone regeneration by responding to mechanical loading through the secretion of signaling molecules such as RANKL and sclerostin [42]. At the same time, cytokines released by osteocytes affect surrounding bone cells and distant organ functions via paracrine and endocrine pathways [43]. Additionally, osteocytes stimulate osteoclastogenesis by releasing molecules such as RANKL, HMGB1, and pro-inflammatory cytokines [44]. However, osteocytes show mechanosensitivity changes and cell death when there are increased levels of pro-inflammatory cytokines [45]. Thus, maintaining the health and function of osteocytes is essential for preserving their integrity.

Osteal macrophages (osteomacs) have an important role in bone formation, reshaping, repair, and renewal. They serve as osteoclast precursors and form bone-resorbing osteoclasts in the presence of RANKL and M-CSF. At the same time, osteomacs are observed localized together with osteoclasts in areas where bone resorption occurs [46,47]. In studies conducted in mouse models, it has been shown that ovariectomy (OVX) leads to the production of osteomacs especially in the postmenopausal period. Additionally, the depletion of osteomacs led to an increased extracellular TRAP (a marker of osteoclast activity) and increased osteoclast levels, directly influencing bone resorption [48].

Extracellular vesicles (EVs) derived from vascular endothelial cells are directly effective in the maturation of osteoblasts and osteoclasts. Studies have shown that the joint use of EVs and curemia is effective in preventing the development of osteoporosis [49]. There are different endothelial cell subtypes that play different roles in the postnatal bone development process, and imbalances in these subtypes accelerate age-related bone loss and affect the development of osteoporosis [50].

As osteoporosis is a complex disease, disruptions in this cycle can result in weaker bones [51]. Non-alterable factors such as gender, age, ethnicity, family history, and body mass index (BMI), along with controllable factors like physical inactivity, low Ca and vitamin D intake, unhealthy diet, smoking, excessive alcohol consumption, and certain medications (such as glucocorticoids), can cause these disruptions. In osteoporosis, the balance in the bone cycle is disrupted, decreasing bone hardness and durability, leading to brittle bones that are more susceptible to fractures (Figure 1B) [52].

### 2.1. Roles of Hormones and Cytokines in Osteoporosis

Among the reasons for the development of osteoporosis, such as genetic predisposition, nutritional problems, aging, chronic diseases, hormonal change, and inflammation [53,54], this section will focus on hormones such as parathyroid, estrogen, testosterone, and calcitonin and the effects of pro-inflammatory and anti-inflammatory cytokines.

#### 2.1.1. Hormones

A decrease in estrogen levels, especially during menopause, is an example of an important hormonal change that enhances the risk of osteoporosis. Studies have emphasized that insufficient estrogen production causes difficulties in maintaining bone density, which in turn leads to increased bone fragility and a higher risk of osteoporosis [55]. In addition, the decrease in estrogen hormone levels during menopause increases the risk of osteoporosis by changing the expression of estrogen target genes and increasing the secretion of inflammatory cytokines [56]. In general, low testosterone levels in men also increase the risk of osteoporosis, as the testosterone hormone helps protect bone health [57]. Additionally, an overactive thyroid gland or thyroid disorders also affect bone turnover, and disorders in the pituitary gland can trigger osteoporosis [54,58]. Overactivity of the parathyroid glands known as hyperparathyroidism, high cortisol levels resulting from conditions such as Cushing’s syndrome [54] changes in progesterone levels during menopause [55], changes in insulin-like growth factor (IGF) 1 levels [54], changes in inhibin B, and decreases in anti-Müllerian hormone levels affect bone health and contribute to the risk of osteoporosis [54]. 

##### Growth Hormone (GH)

GH stimulates bone formation and resorption by both increasing the number and enhancing the function of osteoblasts [59]. GH is produced in the pituitary gland and stimulates insulin-like growth factor 1 (IGF-1) production from the liver and other tissues [60]. The effects of this axis on bone are complex and determined by both systemic and local IGF-1 production and the effects of IGF-binding proteins synthesized by bone cells. In the case of GH or IGF-1 deficiency, osteoblastogenesis is impaired, bone strength is reduced and fracture risk is increased. This is also observed in functional disruptions of the GH-IGF-1 axis, such as through aging or anorexia nervosa, in which BMD and bone microarchitecture parameters are negatively affected independently from BMI [61]. Patients with acromegaly also have impaired bone structure and increased risk of fractures, indicating that GH deficiency and excess are detrimental to skeletal health [62]. 

Despite GH deficiency causing adverse effects on bone structure, GH treatment increases BMD in individuals with osteoporosis and raises bone mass by affecting Ca and phosphate absorption in the intestine [63]. According to a study by Krantz et al., all patients received hormone replacement therapy (HRT) and Ca/vitamin D. Over a 10-year follow-up, the incidence of fractures in patients receiving GH treatment decreased from 56% to 28%, while in the control group, it increased from 8% to 32%. Although this decrease in fracture incidence in the treated group may be associated with increased awareness and new osteoporosis treatments (bisphosphonates, teriparatide), this study provides significant results in evaluating the long-term efficacy of GH treatment in postmenopausal osteoporosis [64].

##### Glucocorticoids

Glucocorticoids contribute to the development of osteoporosis by increasing bone resorption and decreasing bone formation [65]. Glucocorticoids increase the number and lifespan of osteoclasts, promote the production of RANKL, RANK, and IL-6, which enhances osteoclastogenesis while inhibiting the synthesis of osteoprotegerin and interferon-beta (IFN-β) [66]. Additionally, glucocorticoids suppress the production of sex hormones, reducing the inhibitory effect on osteoclasts [55]. In contrast, consuming glucocorticoids results in the inhibition of replication and differentiation in osteoblasts, induces their apoptosis, and suppresses IGF-1 secretion. By reducing the number of osteocytes, glucocorticoids hinder the repair of bone microdamage and compromise bone biomechanical properties. Glucocorticoids also decrease intestinal Ca absorption and increase urinary Ca excretion, although PTH levels do not differ from the controls. Moreover, glucocorticoids cause muscle atrophy and weakness, increasing the risk of falls and, consequently, fractures, with glucocorticoid-induced osteoporosis program (GIOP) patients experiencing more fractures than those with menopause-related osteoporosis. U.S. Food and Drug Administration-approved antiresorptives and bone metabolism drugs are available for GIOP treatment, but evidence of their efficacy in rheumatoid arthritis patients, requiring long-term glucocorticoid therapy, is limited. Excessive glucocorticoids promote differentiation from osteoblasts to adipocytes, interfere with bone morphogenetic protein (BMP) signaling pathways, inhibit osteoblastic differentiation, and increase apoptosis of osteoblasts and osteocytes [29,55,65,66]. Therefore, understanding the impact of glucocorticoids on bone metabolism is crucial for developing more effective therapeutic strategies to mitigate their adverse effects.

##### PTH

PTH is a critical regulator of Ca and phosphate homeostasis. It promotes bone resorption and facilitates the transport of Ca and phosphate from the bones to the bloodstream. Chronic elevations in PTH levels, such as those observed in primary hyperparathyroidism, can precipitate osteoporosis. Chronic elevation of PTH can be caused by conditions such as primary hyperparathyroidism, which is characterized by an overproduction of PTH due to a benign tumor in one or more of the parathyroid glands [67]. Secondary causes of elevated PTH levels include chronic kidney disease, where impaired kidney function leads to phosphate retention and hypocalcemia, subsequently stimulating the parathyroid glands to produce more PTH [68]. PTH stimulates bone resorption by increasing RANKL expression and promoting osteoblast activity. A study examining the effects of PTH on bone metabolism [69] showed that PTH enhances the activity of osteoblasts through the Wnt/beta-catenin signaling pathway. The effects of PTH on bone are complex and not fully understood, because PTH can have both catabolic and anabolic effects on bone [70].

PTH primarily functions through the PTH/PTHrP receptor (PTH1R), a G protein-coupled receptor located on the surface of osteoblasts and kidney cells [71]. Upon binding to PTH1R, PTH activates multiple signaling cascades, notably the cyclic adenosine monophosphate (cAMP)/protein kinase A (PKA) pathway and the phospholipase C (PLC) pathway, which collectively regulate Ca homeostasis and bone remodeling [72]. In osteoblasts, the activation of the cAMP/PKA pathway promotes the expression of genes associated with bone formation, including collagen type I and alkaline phosphatase, while also inhibiting apoptosis, thereby prolonging the lifespan of osteoblasts [72]. Conversely, continuous exposure to PTH shifts the signaling dynamics towards the PLC pathway, increasing the production of inositol trisphosphate (IP3) and diacylglycerol (DAG), which enhances the expression of RANKL [72]. In addition, it is important to emphasize that the intermittent administration of small doses of PTH stimulates osteoblast activity, whereas continuous high-dose PTH treatment leads to the downregulation of genes involved in osteoblast differentiation and function, tipping the balance towards bone resorption. These complex and dual effects underline the hormone’s capacity to affect bone formation and resorption simultaneously, depending on the mode and duration of exposure [73,74,75,76,77].

In short, intermittent PTH administration promotes bone formation by activating osteoblasts and prolonging their lifespan, whereas continuous exposure promotes bone resorption as it leads to the downregulation of osteoblastic activity [78]. This dual action is due to the different signaling pathways activated by PTH under different exposure conditions. Intermittent PTH activates anabolic pathways such as the cAMP/PKA pathway, while continuous PTH exposure activates catabolic pathways, leading to increased RANKL expression and osteoclastogenesis [74,75,76,77,78,79].

##### Estrogen Hormone

Postmenopausal osteoporosis, resulting from estrogen deficiency, is the most common type of osteoporosis [28]. Estrogen works by binding to estrogen receptors (ERs), regulating the expression of proteins such as IL-1, IGF-1, and transforming growth factor-beta (TGF-β) [28]. ERs are highly expressed in osteoblasts, osteoclasts, and osteocytes, and they could block RANKL activity accordingly, preventing osteoclast production and bone resorption [80]. In the case of estrogen deficiency, the expression of these target genes changes and the release of inflammatory factors such as IL-1, IL-6, and TNF increases. Additionally, a study has demonstrated that a lack of estrogen directly impacts apoptosis and cell differentiation [81,82,83,84].

Estrogen also promotes the apoptosis of osteoclasts and inhibits osteoclastogenesis through various pathways. In addition to increasing OPG production, it reduces osteoclast differentiation by suppressing factors such as IL-1 and TNF. In the case of estrogen deficiency, bone resorption and formation become unbalanced and osteoclastic resorption increases, and as a result, further bone loss occurs. In turn, OPG regulates osteoclast activity by contributing to the anti-resorptive effects of estrogen [84,85]. 

To reduce the effect of the estrogen hormone on the development of osteoporosis, HRT is often used to balance estrogen levels, minimizing bone loss and reducing the risk of osteoporosis by mimicking the effects of estrogen and progesterone, leading to increased bone resorption and decreased bone formation [86]. This therapy is especially useful for women who are going through menopause or have premature ovarian failure because it can effectively reduce the risk of osteoporosis-related spine and hip fractures [87].

##### Testosterone Hormone

The testosterone hormone, in addition to being the main sex hormone in men, has an important role in bone health, which also exists in women. It signals through the androgen receptor, which is predominantly expressed in osteoblasts and osteocytes [88]. Low testosterone levels in men and women compared to their reference values for each gender have been associated with osteoporosis [89]. The influence of testosterone on bone health can be explained in complex interactions that occur at the cellular and molecular level. Testosterone stimulates bone formation by increasing the differentiation and activity of osteoblasts [90]. It also inhibits the activity of osteoclasts to prevent bone destruction, then suppresses the activity of IL-6, which activates osteoclasts and promotes bone resorption. In contrast, testosterone deficiency promotes the activation of RANKL production from osteoblasts, which contributes to the promotion of differentiation and functions in osteoclasts while increasing bone resorption [91,92]. Additionally, testosterone deficiency reduces BMD through increased IL-6 activation [93]. Considering these overall mechanisms, testosterone directly reduces the risk of osteoporosis by increasing bone density and strength while indirectly affecting the bone metabolism through GFs [91,92,93,94,95].

A recent study suggests that testosterone replacement therapy is a potential strategy for preventing and treating osteoporosis that significantly increases cortical volumetric BMD (3%) and areal BMD at the lumbar spine and hip [96]. However, the balance between the potential benefits and risks of testosterone therapy are important. The risks of testosterone-replacement therapy should be considered, especially in the presence of hormonal-dependent malignancies such as prostate cancer. In a systematic review and meta-analysis of testosterone therapy, a significant improvement was observed in spinal BMD and in the hypogonadal population [97,98]. As a result, the positive effects of the testosterone hormone on bone health play an important role in the pathogenesis of osteoporosis. Maintaining optimal testosterone levels is a critical factor in preventing osteoporosis by increasing bone density and reducing the risk of fractures [1].

##### Calcitonin Hormone

Calcitonin, a hormone produced by the parafollicular cells of the thyroid gland, regulates Ca metabolism. It reduces bone resorption by inhibiting osteoclast activity, contributing to the maintenance of BMD and reducing the risk of bone fractures [99]. Consequently, it is used to treat bone disorders such as osteoporosis, hypercalcemia, and Paget’s disease.

Since its discovery over 50 years ago [100], the preventive effect of calcitonin on bone resorption has been extensively studied. Notably, Ca metabolism or BMD is unaffected in patients with chronically elevated calcitonin levels or in those with calcitonin deficiency after thyroidectomy [101,102]. In light of this research, the physiological function of calcitonin is questioned. Some studies propose that calcitonin lacks a biological or functional role in mammals [103,104,105]. Conversely, there is evidence suggesting that calcitonin plays a crucial role in protecting the skeleton under Ca stress conditions. Numerous investigations revealed the effects of calcitonin on osteoblast division and bone formation. A recent study showed that osteoblast division and bone formation were aided by calcitonin therapy. It impedes the process of bone development even after the bone production process is finished [106]. Subsequent studies found that calcitonin has a rapid stimulating effect on osteoblast proliferation [107,108]. However, calcitonin did not alter the indices of bone growth in adult mice that received a local injection of calcitonin, nor did it exhibit any effect on osteoblastic proliferation in rats [109,110].

In conclusion, the relationship between calcitonin and skeletal biology is complex and not fully understood. While the role of calcitonin in preventing bone resorption is clear, its physiological function and role in Ca homeostasis or bone disorders in mammals remain contentious.

#### 2.1.2. Cytokines

The term cytokine was first introduced to the literature by Cohen and colleagues in 1974 [111]. Cytokines are composed of a family of proteins and glycoproteins secreted by cells, exerting specific effects on interactions and communications between cells [112]. Initially, these molecules were believed to originate solely from lymphocytes, leading to the designation of lymphokines. Subsequently, it was recognized that monocytes also produce these factors, resulting in the term monokine. However, it is now understood that these mediators are not exclusively secreted by lymphoid cells, hence the more prevalent use of the term cytokine [113].

The molecular weight of cytokines ranges between 5 and 20 kDa, serving as messenger molecules of the immune system. Although cytokines are mainly synthesized and secreted by macrophages and leukocytes, their production can also be through endothelial cells and fibroblasts. These molecules play critical roles in coordinating the functioning of the immune system and defense against infections while also having an impact over various other cell types such as those in the nervous and endocrine systems [113,114,115].

**Figure 2:**
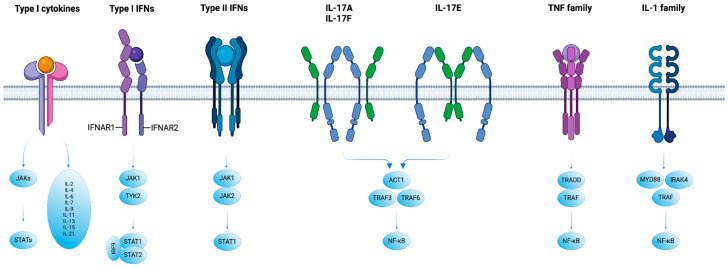
Cells expressing cytokine receptors. This figure was created using https://app.biorender.com/, accessed on 18 June 2024.

Cytokines, which include lymphokines, monokines, chemokines, and interleukins, can act through autocrine, paracrine, or endocrine mechanisms and exhibit pleiotropy and redundancy in their functions (Figure 2). They are produced in a cascade manner, often stimulating target cells to produce additional cytokines and can act synergistically or antagonistically [114].

Cytokines serve as mediators and modulators in various local environments, regulating immune responses, hematopoietic development, and intercellular communication [116]. Additionally, they govern host responses to infectious agents and inflammatory stimuli. Cytokines can exhibit additive, synergistic, or antagonistic effects through interactions with each other [117]. Occasionally, one cytokine may induce or modify the production of another. The pleiotropic nature of cytokines is associated with their ability to influence multiple targets and direct various physiological effects. These characteristics demonstrate that cytokines have a broad impact on various biological processes [114,118,119]. 

Various immune cells and tissues, including macrophages, B lymphocytes, T lymphocytes, mast cells, endothelial cells, fibroblasts, and various stromal cells, are also involved in bone metabolism. Cytokines produced by these cells are effectively regulated in processes such as bone resorption and formation, and a specific cytokine can be synthesized by multiple cell types [120].

Cytokines are typically grouped into two main categories: pro-inflammatory and anti-inflammatory cytokines [10,115]. In the case of infection or tissue damage, pro-inflammatory cytokines are produced primarily by activated macrophages and other immune cells. These molecules are effective in initiating and sustaining inflammation, thereby enabling the immune system to effectively combat pathogens [121,122]. On the other hand, anti-inflammatory cytokines are molecules designed to control and limit the inflammatory response. They are generally produced to prevent the excessive intensification of inflammation. By suppressing the effects of pro-inflammatory cytokines and balancing the immune response, anti-inflammatory cytokines assist in controlling tissue damage and inflammation [123,124].

**Figure 3:**
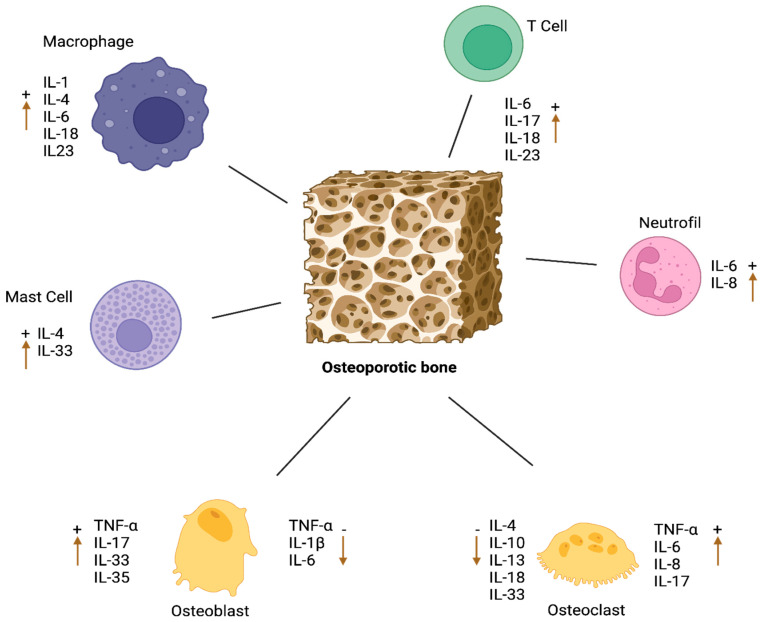
The upregulation and downregulation of cytokines in different cells during osteoporosis. (TNF-α can exhibit both upregulation and downregulation.) This figure was created using https://app.biorender.com/, accessed on 18 June 2024.

The complex interactions and balancing of cytokines play a critical role in maintaining a healthy immune response and tissue homeostasis (Figure 3). Inflammatory processes rely on the delicate balance between pro- and anti-inflammatory cytokines. As a result, the pro-inflammatory and anti-inflammatory properties of cytokines work together to maintain the health of the immune system and provide an effective defense mechanism against pathogens. Table 1 shows the effects of cytokines on osteoporosis.

The BMU is a functional unit in bone remodeling, forming transient structures in microscopic areas. These units are characterized by the resorption and formation activities of cells such as osteoclasts and osteoblasts. BMUs play a critical role in the continuous renewal and repair of bone tissue. The process incorporates several phases: in the resorption phase, osteoclasts resorb old or damaged bone tissue; in the reversal phase, post-osteoclastic cells clean the surface; in the formation phase, osteoblasts create and mineralize a new bone matrix; and in the termination phase, osteoblasts complete the process by forming a matrix on the bone surface [125]. Additionally, postmenopausal estrogen level decline increases BMU activity, leading to bone loss. In osteoporosis, the resorption cavities created by BMUs are not fully filled, increasing bone porosity and weakening the bone tissue. Thus, osteoporosis treatments target BMUs to reduce bone loss and increase bone density [38,125,126,127,128].

##### Pro-Inflammatory Cytokines

TNF-alpha (TNF-α)

TNF-α is defined as an inflammatory cytokine, an essential component of the immune system. This factor plays a pivotal role in inflammation processes and is released by various cells, exerting diverse biological effects. Particularly, TNF-α plays a critical role in the immune system’s response to infections and tissue repair [129,130]. The effects of TNF-α on bone metabolism are particularly significant as this cytokine directly influences the structure of bone tissue. TNF-α stimulates bone resorption by increasing the activation of cells called osteoclasts, leading to bone degradation. Since TNF-α inhibits the function of osteoblasts, thereby impeding bone formation, excessive secretion of TNF-α or chronic inflammation can result in decreased bone density and bone loss. In this context, the impact of TNF-α on bone health is especially pertinent to the development of bone diseases such as osteoporosis [131,132]. Overproduction of TNF-α in chronic inflammatory conditions or autoimmune diseases can adversely affect bone health and elevate the risk of osteoporosis [133]. Another important characteristic of TNF-α is initiating and regulating inflammation. This cytokine supports the body’s defense mechanisms against infections and foreign substances. Additionally, the pro-inflammatory effects of TNF-α enhance cellular immune responses by increasing the activation of the immune system. The ability of TNF-α to induce apoptosis (programmed cell death) is also significant for combating infections and tissue repair. However, excessive TNF production or activation may be associated with pathological conditions such as autoimmune diseases, chronic inflammatory conditions, and tissue damage. It is known that TNF-α increases bone resorption in the context of bone metabolism. This plays a significant role in the pathogenesis of diseases associated with bone loss, such as osteoporosis [134,135,136,137,138,139,140].

Finally, the use of TNF-α as a target in the treatment of various immunological and inflammatory diseases is noteworthy. Anti-TNF drugs, for example, are commonly used in the treatment of autoimmune and inflammatory diseases such as rheumatoid arthritis, Crohn’s disease, and psoriasis [141]. These drugs block the biological effects of TNF-α, thereby reducing inflammation and alleviating disease symptoms [141,142]. In conclusion, TNF-α is a critical cytokine for regulation of the immune response and biological effects. However, excessive production or activation of TNF-α can contribute to the development of various pathological conditions, emphasizing the importance of understanding its biological effects and regulation. A better understanding of these effects may lead to the development of new strategies for the treatment and prevention of bone diseases.

IL-1

IL-1 is a class of cytokines that is also active in bone health, encompassing IL-1α and IL-1β [114]. IL-1 serves as a significant mediator of inflammatory bone loss by influencing osteoclast differentiation, multinucleation, and cell survival processes. Particularly, IL-1β acts as a determinant factor in promoting osteoclast formation and enhancing the production of RANKL. Thus, it supports osteoclastogenesis in conjunction with the effects of TNF-α and can trigger osteoclast precursor differentiation by increasing RANKL expression in stromal cells [11,143,144,145]. IL-1 can initiate osteoclast differentiation through RANKL/RANK-independent activation in bone marrow-derived macrophages (BMMs). Furthermore, IL-1, in conjunction with TNF, triggers bone resorption in osteoblasts, leading to bone loss. It may also negatively impact fracture healing processes by inhibiting human osteoblast migration. However, IL-1α can promote the formation of osteoclast-like cells by increasing the expression of M-CSF and prostaglandin 2 (PGE2) while reducing OPG expression in osteoblasts [11,146].

Clinical data regarding the immune phenotype of postmenopausal patients indicate elevated levels of inflammatory cytokines in postmenopausal women, both in peripheral blood cells and directly in the bone microenvironment [147,148,149]. Particularly, a relationship between bone resorption and increased IL-1 levels has been established, which can be prevented with estrogen therapy. Preclinical data suggest increased levels of inflammatory mediators and changes in immune cell profiles in rodents with estrogen deficiency [147,148,149,150]. These alterations lead to the emergence of a chronic low-grade inflammatory phenotype, summarizing most of the clinical characteristics of the immune phenotype in postmenopausal osteoporosis.

To further investigate the effect of IL-1 in osteoporosis, Harrell et al. aimed to assess the immunosuppressive effects of mesenchymal stem cells (MSCs) and the potential of the IL-1 receptor antagonist (IL-1Ra). They demonstrated that MSCs suppress inflammation by secreting IL-1Ra, exhibit anti-inflammatory effects, and contribute to tissue healing. However, it is noted that IL-1Ra may increase the risk of infection, and MSCs producing IL-1Ra may be contraindicated in patients with immunodeficiencies or a history of recurrent infections. In conclusion, while the therapeutic potential of MSC-derived IL-1Ra is emphasized, further research and clinical studies are needed to determine dosage and application methods [151]. Another study by Yu et al. investigated the effect of oridonin (ORI) on osteoporosis induced by estrogen deficiency in mice and confirmed its anti-osteoporotic effect through mouse experiments. The research demonstrated that ORI improves bone health in mice by reducing osteoporosis and regulating cytokines involved in the bone-remodeling process, while also enhancing immunity. In this process, cytokines such as TNF-α, IL-1, IL-6, and IGF-1 were found to play a significant role [152].

In conclusion, IL-1 represents a class of cytokines with significant effects on osteoclast differentiation and bone resorption. Particularly, increased IL-1 levels in postmenopausal osteoporosis are considered a fundamental driver of bone loss, which can be controlled with estrogen therapy [149,150,151,152,153].

IL-6

IL-6 is a member of the cytokine family, an essential component of the immune system. This family includes cytokines such as IL-11, Oncostatin M (OSM), leukemia inhibitory factor (LIF), and cardiotrophin-1 (CT-1) [11,154]. IL-6 is produced by various cell types, particularly abundant in leukocytes such as monocytes, macrophages, T cells [155], and neutrophils [156]. There are two main receptor mechanisms to transmit the biological effects of IL-6: a membrane-bound IL-6 receptor (IL-6R) and soluble IL-6 receptor (sIL-6R) [154,155,156,157]. Activation of these receptors initiates a variety of biological responses by triggering various cellular signaling pathways.

The effects of IL-6 on bone metabolism are highly diverse. A particularly noteworthy response is the recognition of IL-6 as a pro-osteoclastic factor that supports osteoclastic processes. For example, in mouse models overexpressing IL-6, phenotypes such as increased osteoclasts and decreased bone trabecular volume are observed [158,159]. Furthermore, it has been recorded that IL-6 can stimulate osteoclast formation independently from RANKL and indirectly increase osteoclastogenesis by stimulating the RANKL produced by osteoblasts [160]. However, IL-6 has also been found to contribute to the osteogenesis process. Specifically, IL-6 causes an increase in osteogenic capacity marked by increases in osteogenic gene expression. However, it is known that IL-6 can inhibit osteoblast differentiation and increase bone resorption [161,162].

Understanding the biological effects of IL-6 may help to discover novel targets in the treatment of osteoporosis. Modulation of IL-6 signaling pathways or the use of neutralizing antibodies against IL-6 may reverse osteoporotic phenotypes by regulating bone metabolism [163,164]. Additionally, the role of IL-6 in the immune system is also significant, since IL-6 regulates communication between immune cells and initiates the inflammatory response during the inflammation process [124,149]. It has been suggested that the excessive production or chronic inflammation of IL-6 may lead to adverse health issues [160]. For example, autoimmune diseases such as rheumatoid arthritis may arise due to overproduction of IL-6. It has also been suggested that IL-6 may play a role in the development of conditions such as cancer metastasis, atherosclerosis, and type 2 diabetes. However, research on the effects of IL-6 on the nervous system indicates its potential role in processes such as learning and memory [129,154].

Many researchers study the influence of IL-6 in osteoporosis [165,166,167]. For example, Coates et al. investigated the role of IL-6 in full and stress fracture repair models. Following full femur fracture, there were no differences observed in callus morphology and mineral density between IL-6 knockout (KO) mice and control mice. The results indicated that IL-6 KO does not alter the recruitment of immune cells or osteoclast numbers in the stress fracture callus and Wnt1 expression is significantly higher in the IL-6 KO stress fracture callus at both day 1 (KO: 12.5-fold, WT: 5.7-fold) and day 3 (KO: 4.7-fold, WT: 1.9-fold). In conclusion, IL-6 KO enhances callus and bone formation following stress fracture injury, potentially directly impacting the osteoblasts’ ability to produce bone tissue, suggesting a suppressive role of IL-6 in intramembranous bone formation [168]. Another study by Wang et al. investigated the effect of IL-6 on BMP9-triggered osteogenic differentiation in mouse embryonic fibroblasts (MEFs). The findings indicate that BMP9 enhances osteogenic markers and bone formation in conjunction with IL-6, whereas its absence inhibits this process. The relationship between inflammation and bone metabolism was investigated, suggesting that BMP9 may augment osteogenic potential by increasing inflammation. It is noted that IL-6 regulates BMP9 osteogenic ability through the phosphoinositide 3 kinase (PI3K)/Akt/mammalian target of the rapamycin (mTOR) pathway. Furthermore, it is proposed that IL-6 activates the signal transducer and transcription (STAT) 3/mammalian target of rapamycin complex (mTORC) 1 signaling pathway to support BMP9’s osteogenic potential. These findings suggest that the moderate stimulation of inflammation may serve as a significant mechanism to accelerate bone formation facilitated by IL-6 [169].

Studies on supplementary treatments show that they also have an impact on IL-6 levels. For instance, Kassem et al. found that treatment with 0.01 to 10 nM 17β-estradiol, which is a form of estrogenic hormone, does not affect the structural production of IL-6. However, stimulated production induced by IL-1β plus TNF-α was dose-dependently reduced to 74% of the control (*p* < 0.01). This effect was blocked by treatment with type II antiestrogen ICI 182,780. Treatment with hydrocortisone reduced IL-6 production to 17% of control (*p* < 0.001). According to Northern analysis, treatment with 0.01–10 nM 17β-estradiol dose-dependently decreased IL-6 mRNA levels. These findings suggest that at least part of estrogen’s antiresorptive effect may occur through the reduction of IL-6 production in osteoblastic cells [170]. Following this, Zhang et al. investigated the antiosteoporotic effect of hesperidin supplementation in an ovariectomized rat model of osteoporosis. Hesperidin was orally administered to OVX rats at doses of 5, 10, and 20 mg/kg for 10 weeks. The results demonstrate that hesperidin treatment reduces body weight and increases uterine weight in OVX rats, along with significant alterations in biochemical parameters (alkaline phosphatase (ALP), osteocalcin, acid phosphatase, β-isomerized C-terminal telopeptide). Additionally, hesperidin enhances BMD, improves biomechanical parameters, and reduces oxidative stress. Hesperidin is observed to decrease cytokine levels and inflammatory mediators while increasing levels of Ca, P, and vitamin D. Collectively, the protective effect of hesperidin against osteoporosis is attributed to its ability to enhance not only biomechanical parameters but also bone density and mineral content [171].

In conclusion, IL-6 plays a significant role in many biological processes such as the immune system and bone metabolism. Understanding the functions of IL-6 allows for the development of new strategies for the treatment and prevention of osteoporosis.

IL-17

IL-17 is a cytokine secreted by Th17 cells and plays a crucial role in bone metabolism. Th17 cells, a subset of T cells, participate in regulating the immune response, and the secretion of IL-17 is also associated with the pathogenesis of various inflammatory and autoimmune diseases [11,172]. In conditions like estrogen deficiency, an increase in IL-17 levels exhibits bone loss-promoting effects. For instance, enhancing osteoclastic activity results in an increase in bone resorption, leading to decreased bone density and an increased risk of fractures [173,174].

Anti-IL-17 therapies can alleviate symptoms of bone loss and support new bone regeneration in osteoporotic fractures. However, the effects of IL-17 vary depending on concentration [172,173]. High IL-17 concentrations can inhibit the differentiation of osteoclast precursors, thereby reducing bone resorption. However, high IL-17 levels can also negatively impact bone regeneration processes by promoting the early differentiation of osteoblasts [175].

It has been demonstrated that in autoimmune diseases such as rheumatoid arthritis, IL-17 exacerbates bone destruction. IL-17 stimulates osteoclast formation by increasing RANKL expression, thus promoting bone erosions. This condition can negatively affect bone health and play a significant role in the pathogenesis of rheumatoid arthritis [176]. In cases of estrogen deficiency, activation of the Th17 cell subset can increase bone loss. It has been shown that IL-17 triggers bone loss by increasing osteoclast formation and the expression of pro-osteoclastogenic cytokines by osteoblasts. Therefore, inhibiting IL-17 could be a potential therapeutic approach for preserving bone health. Overall, IL-17 is a significant factor in regulating osteoclastic and osteoblastic processes. Better understanding of these mechanisms may help identify new targets for the treatment of bone diseases such as osteoporosis. Further research into the role of IL-17 in bone metabolism and the development of therapeutic interventions targeting IL-17 may be beneficial for maintaining bone health [11,147,174,177,178,179,180].

IL-8

IL-8 emerges as a cytokine synthesized by osteoblasts and supports RANKL-induced osteoclastogenesis. The effect of IL-8 can be inhibited under in vitro conditions by anti-IL-8 antibodies or IL-8 receptor inhibitors [129,181]. While human osteoclasts produce IL-8, current research still presents gaps regarding this cytokine. One reason for these gaps is the absence of an IL-8 equivalent cytokine in rodents, complicating the modeling of human diseases [11]. In this context, discovering an effective IL-8 homolog in rodents could be a significant guiding factor for future IL-8 research [182]. The effects of IL-8 on bone metabolism, particularly in bone diseases such as postmenopausal osteoporosis, raise interest in its pathophysiology. In postmenopausal women, increased bone resorption and consequent decreased bone density have been associated with a significant increase in IL-8. This suggests that IL-8 actively participates in bone metabolism by promoting osteoclast activation [183]. The effects of pharmacological interventions on IL-8 have been studied, showing that certain drugs like atorvastatin reduce IL-8 levels and thus cause bone loss in glucocorticoid-induced osteoporosis models [183]. These findings suggest that the role of IL-8 in bone metabolism could be targeted pharmacologically. Inflammatory conditions like chronic obstructive pulmonary disease (COPD) can induce changes in bone metabolism associated with IL-8. Increased plasma IL-8 levels have been observed in COPD patients, correlated with neutrophil RANKL expression. These findings indicate that IL-8 increases plasma levels and is associated with an increase in RANKL neutrophils in COPD patients, while also suggesting the systemic inflammatory environment may be a mechanism driving RANKL expression by neutrophils in COPD. Neutrophils, as RANKL-producing cells, are suggested to increase COPD-associated bone loss by participating in osteoclastogenesis. These findings highlight the importance of IL-8 in bone metabolism and suggest targeting IL-8 as a potential strategy for treating or preventing bone diseases. However, considering the complex and bidirectional effects of IL-8, further research and clinical trials are needed for IL-8-targeted therapeutic interventions [11,129,184].

IL-18

Interferon-gamma inducing factor (IGIF), or commonly known as IL-18, is a proinflammatory cytokine with various biological functions [11,185]. Similar to IL-12, IL-18 plays a significant role in regulating various immune responses in the body. Particularly in Fas signaling induced with TNF-α, IL-18 can inhibit osteoclast formation by triggering apoptosis in myeloid cells. However, studies have shown that anti-Fas ligand (FasL) antibodies do not completely block this apoptosis. In the presence of TNF-α, IL-12 and IL-18 synergistically increase nitric oxide (NO) production, thereby promoting cellular apoptosis. Additionally, IL-18 can indirectly inhibit osteoclast formation by stimulating the production of IFN-gamma (IFN-γ) and granulocyte-macrophage colony-stimulating factor (GM-CSF) in T cells [186,187,188,189,190,191].

IL-18 binding protein (IL-18BP) exhibits an antagonistic effect against IL-18 and possesses anti-inflammatory properties. Studies on mice have demonstrated that treatment with IL-18BP prevents bone loss. In osteoporotic women, it has been observed that while IL-18 levels are rising up, IL-18BP levels are decreasing [190]. These findings suggest that employing IL-18BP could be a potential strategy in the treatment of postmenopausal osteoporosis.

On the other hand, other proinflammatory cytokines like IL-6 also promote osteoclast formation [192,193]. IL-18 is secreted through macrophages to regulate the differentiation of Th1 cells and contributes to the inhibition of TNF-α-mediated osteoclastogenesis [194].

Bone loss and fracture risk associated with celiac disease may stem from the increased reactivity of T cells in the intestinal mucosa to autoantigens like gliadin [195,196]. In untreated celiac patients, an increase in bone resorption markers is associated with elevated IL-6, decreased levels of IL-12 and IL-18, and an increase in the RANKL/OPG ratio. This suggests that celiac disease may lead to cytokine imbalance affecting osteoclast and osteoblast activities [197,198].

Finally, alongside cytokines that promote osteoclast development, factors such as IL-4, IL-10, IL-18, and IFN-γ are known to inhibit osteoclast formation.

IL-23

IL-6 and IL-23, a member of the IL-12 family, are cytokines that play a significant role in bone metabolism. IL-23 promotes the transformation of naive CD4(+) T cells into Th17 cells, thereby increasing the production of IL-17 by Th17 cells [155]. This mechanism contributes to T cell-mediated osteoclast formation and is known as the IL-23/IL-17 axis. Studies with mouse models have observed less bone loss in inflammatory bone destruction models of mice lacking IL-17 or IL-23 [178]. However, the role of IL-23 in bone metabolism is not fully understood; some research suggests that IL-23 may indirectly inhibit osteoclast differentiation in CD4(+) T cells [199].

Cytokines such as IL-6, IL-18, IL-23, IL-27, and TNF-α released by macrophages can influence osteoclast activation and differentiation [177]. For example, proinflammatory cytokines like IL-6 and TNF-α can increase osteoclast differentiation and activation [200,201], while cytokines like IL-27 and IL-18 can inhibit this process [178,202]. Moreover, macrophages are responsible for the release of factors that not only promote osteoclastogenesis but also inhibit osteoblast apoptosis and stimulate new bone formation. Therefore, macrophages play a critical role in balancing bone metabolism [11,192,202].

In conclusion, the role of immune cells such as IL-23 and macrophages in bone metabolism is complex and still under research. These cells and the cytokines they secrete regulate osteoclast and osteoblast activities, thereby affecting the bone remodeling process.

IL-33

IL-33 is receiving increasing attention as a cytokine that regulates bone metabolism and is involved in the pathogenesis of osteoporosis [129,203]. IL-33 is the newest member of the IL-1 family and acts as both an intracellular and extracellular alarm [133,204]. Human IL-33 is secreted from non-hematopoietic cells such as endothelial and epithelial cells and functions as a ligand for the orphan receptor ST2 of the Toll-like receptor (TLR)/IL1R superfamily [205,206].

It has been observed that postmenopausal women with osteoporosis have lower levels of IL-33 compared to healthy control women [207]. IL-33 helps maintain bone health by inhibiting osteoclast formation and increasing osteoblast function. Particularly, it is known to suppress RANKL-induced osteoclast formation by interacting with its specific receptor ST2. This interaction regulates the expression of BLIMP-1 and interferon regulatory factor-8 (IRF-8), thereby inhibiting the expression of the nuclear factor of activated T cells 1 (NFATc1) and preventing osteoclast formation. Additionally, IL-33 prevents osteoporosis by increasing apoptosis in osteoclasts and directly affecting osteoblasts [11,207,208].

The biological effects of IL-33 are associated with type 2 immune responses. Th2 cells, eosinophils, mast cells, basophils, and group 2 innate lymphoid cells (ILC-2) respond to IL-33 activation [204,205]. It has been observed that systemic levels of IL-33 increase during inflammation, which is significant in conditions such as allergic and autoimmune diseases [205,209]. Following clinical studies have been conducted to better understand the role of IL-33 in osteoporosis. For example, it has been suggested that low IL-33 levels in postmenopausal osteoporotic women could act as a potential diagnostic marker for the treatment of bone loss [13]. These potential effects of IL-33 in maintaining bone health represent a promising area for the treatment of osteoporosis and other bone diseases.

In conclusion, the role of IL-33 in bone metabolism is considered a significant factor in the pathogenesis of osteoporosis. However, the complex interactions and molecular mechanisms of IL-33 require further research.

##### Anti-Inflammatory Cytokines

IL-10

IL-10 is a cytokine that exerts a significant impact on bone metabolism, particularly in osteoporosis. The role of IL-10 in preserving and regulating bone health occurs through a series of complex mechanisms. Specifically, it is observed that IL-10 regulates both osteoclast and osteoblast activities to maintain bone balance [11,114,129,210].

When examining the effects of IL-10 on osteoclast activity, it has been observed that this cytokine inhibits osteoclast differentiation and reduces bone resorption. Studies in mouse models have demonstrated that IL-10 deficiency leads to decreased bone mass and increased fragility. It has been found that IL-10 promotes bone formation by inhibiting osteoclast differentiation. On the other hand, the effects of IL-10 on osteoblast activity are also significant. IL-10 suppresses osteogenic differentiation by inhibiting bone mineralization and the synthesis of bone proteins. Studies conducted on mouse bone marrow cells have shown that IL-10 is an important regulator of osteogenic activity. The effects of IL-10 on bone metabolism occur not only at the cellular level but also through immunological mechanisms [211]. Particularly, the immunomodulatory effects of IL-10 can regulate inflammatory responses in osteoporosis pathogenesis, affecting bone health. Studies in mouse models have shown that IL-10 deficiency accelerates osteoporosis development and increases bone resorption [212,213].

In conclusion, the effects of IL-10 on bone metabolism are highly complex and play a crucial role in preserving and regulating bone health. Therefore, evaluating IL-10 as a potential therapeutic target in understanding and treating bone diseases such as osteoporosis is of great importance. Future studies are expected to contribute to a better understanding of the effects of IL-10 on bone health and its integration into clinical practices.

IL-4

IL-4, a pleiotropic cytokine, is primarily produced by Th2 cells, mast cells, and eosinophils, which are the key components of the immune system. It regulates immune responses and is recognized as a potent osteoclastogenesis inhibitor, directly and indirectly affecting osteoclast formation and function [214]. 

The endothelial cells within the bone’s vascular system release substances such as osteogenic cytokines and hormones, which play a crucial role in regulating bone growth, restructuring, and healing processes. IL-4 and IL-13 indirectly hinder the formation of osteoclasts by stimulating endothelial cells to produce OPG, a hormone that protects against bone loss. Furthermore, IL-4 demonstrates a collaborative impact alongside other cytokines to impede the generation of osteoclasts [11,215].

IL-4 also possesses pro-regenerative properties and is a cytokine involved in bone metabolism [216]. In macrophages, IL-4 signaling induces M2 macrophage polarization and increases the expression of osteogenic factors. IL-4 inhibits osteoclastogenesis by downregulating RANKL expression and upregulating OPG expression in osteoblasts. Furthermore, IL-4 inhibits bone resorption and enhances the activity and differentiation of osteoblasts [216,217,218].

Th2 cells, produced in the presence of IL-4, exhibit anti-inflammatory activity. Th2 signature cytokines are associated with the inhibition of osteoclastogenesis and can prevent bone loss [179,219,220,221]. The literature provides evidence that Th2 lymphocytes have an osteoprotective role in the pathophysiology of osteoporosis. Th2 cytokines such as IL-31 and IL-33 also regulate bone metabolism and can influence the progression of osteoporosis. While IL-31 enhances osteoclast differentiation, IL-33 inhibits osteoclast differentiation and enhances osteoblast activity [203,222].

These findings highlight the significant role of IL-4 and other Th2 cytokines in bone metabolism, suggesting their potential as therapeutic targets for osteoporosis.

IL-13

IL-13 is a cytokine that constitutes a significant component of the immune system, and the discovery of its roles in bone metabolism in recent years has increased attention to this cytokine. While initially associated with the immune system, its effects on bone tissue are increasingly being elucidated [218,223]. The effects of IL-13 on bone metabolism are particularly focused on the balance between osteoclasts and osteoblasts [217]. Osteoclasts, responsible for bone resorption, have been shown to be potentially inhibited by IL-13, thereby reducing bone loss. Moreover, it is known that IL-13 also affects osteoblasts, thereby promoting bone formation [224,225].

The impact of IL-13 on osteoclasts occurs through the regulation of osteoclast differentiation and activation. IL-13 can target specific signaling pathways in osteoclasts to inhibit this process, potentially contributing to the control of bone resorption and preservation of bone mass. On the other hand, the effect of IL-13 on osteoblasts occurs by increasing the release of factors that promote bone formation. Osteoblasts, responsible for bone tissue formation and renewal, may benefit from the effects of IL-13 in increasing bone formation and maintaining bone tissue health [192,217].

The role of IL-13 in bone metabolism is also associated with the effects of inflammation and immune responses on bone health [226]. For instance, inflammation has been linked to bone loss, and anti-inflammatory cytokines such as IL-13 are thought to play a protective role in this process [227]. However, the exact role of IL-13 in bone metabolism remains incompletely understood, and further research is warranted.

IL-35

IL-35 belongs to the IL-12 cytokine family, representing a novel class of factors with anti-inflammatory and immunosuppressive properties. In bone immunology, IL-35 notably inhibits osteoclast formation and prevents TNF-induced osteoclastogenesis and bone resorption both in vitro and in vivo. These effects are mediated by inhibiting nuclear factor kappa B (NF-κB) and mitogen-activated protein kinase (MAPK) pathways, thereby suppressing NFATc1, c-Fos, and TRAP, while also blocking TNF-induced osteoclast formation and supporting apoptosis via Janus kinase (JAK) 1/STAT1 activation [11,228]. 

The imbalance between bone and adipogenesis is significant in osteoporosis [229]. MSCs have the capability to differentiate into both osteoblasts and adipocytes [230]. IL-35 enhances the proliferation of these cells while inhibiting their apoptosis and adipogenic differentiation. Additionally, by upregulating the expression of key factors in osteoblast differentiation, such as β-catenin and Axin2, IL-35 controls the balance between osteogenic and adipogenic differentiation, suggesting potential applications in the treatment of osteoporosis and obesity [230,231].

Furthermore, IL-35 may play a role in osteoclasts induced by RANKL and M-CSF via the Th17/IL-17 axis, exhibiting inhibitory effects on both metabolic processes associated with osteoporosis [231,232,233]. Recent research demonstrates the physiological and pathophysiological roles of IL-35 in various cells and tissues. IL-35 is essential for bone formation and resorption [233]. Acting on both osteoblasts and osteoclasts, this cytokine induces biological changes in C3H10T1/2 cells, a cell model used to study osteogenesis and adipogenesis in bone marrow. IL-35 has been shown to support osteogenesis and inhibit adipogenesis in MSCs, potentially contributing to crosstalk between the Wnt/β-catenin and peroxisome proliferator-activated receptor γ (PPARγ) signaling pathways [234,235]. These studies contribute to our understanding of the importance of IL-35 in bone health and its potential therapeutic applications.

**Table 1:** Effects of cytokines on osteoporosis.

Cytokine	Family	Effects on Osteoblast	Effects on Osteoclasts	Effects on Osteocytes	Increase	Decrease	References
TNF-α	TNF-α	Osteoblasts are stimulated to produce RANKL and M-CSF. At low concentrations, these molecules enhance the differentiation of mesenchymal precursor cells into osteoblasts, whereas at high concentrations, they impede osteoblast function and halt bone formation. Additionally, the suppression of IGF-1 and Runt-related transcription factor 2 (RUNX2) expressions inhibits osteoblast differentiation.	Stimulates osteoclast development and enhances RANK expression in osteoclast precursors. Supports osteoclast formation via RANKL signaling. Increases c-Fos expression in osteoclast precursors.	TNF-α is known to directly increase RANKL expression in osteocytes and induce osteoclast formation both in vitro and in vivo. Additionally, TNF-α has been observed to upregulate sclerostin expression in osteocytes, which subsequently leads to an increase in RANKL expression.	Bone resorption, osteoclast differentiation and activation	It reduces bone formation by inhibiting osteoblast function.	[131,132,236,237]
IL-1α	IL-1	-	Promotes osteoclastogenesis.	IL-1α promotes osteoclast formation by increasing RANKL expression in osteocytes. Additionally, IL-1α supports the survival of osteocytes and plays a significant role in osteogenesis. IL-1α interacts with osteocytes through complex pathways such as Ca and NO signaling, affecting the balance of bone formation and resorption.	M-CSF, PGE2, bone loss, osteoclast.	OPG	[11,146,238,239,240,241]
IL-1β	IL-1	Induces bone resorption in osteoblasts by activating p38 MAPK. Inhibits human osteoblast migration.	Activates osteoclasts and promotes osteoclast maturation, facilitating multinucleation and viability.	IL-1β influences bone metabolism by increasing sclerostin secretion in osteocytes and inducing osteocyte apoptosis. Furthermore, IL-1β has been reported to enhance osteocyte-mediated osteoclastogenesis, an effect that can be mitigated by mechanical loading. IL-1β promotes the differentiation and mineralization of mesenchymal stem cells into osteoblasts, can increase inflammation in the bone microenvironment, and stimulates the production of FGF23.	RANKL, bone loss, osteoclast.	Bone formation rate.	[149,153,241,242,243,244,245,246]
IL-4	Th2	-	Suppresses osteoclast formation both directly and indirectly, inhibiting the bone resorption activity of mature, differentiated osteoclasts.	IL-4 and IL-10 are key regulators in the interactions between osteocytes and bone health. IL-4, together with IL-10, promotes the osteogenic differentiation of osteoblasts, induces an anti-inflammatory phenotype in macrophages, and inhibits osteoclastogenesis. These cytokines play crucial roles in supporting bone formation and maintaining the balance between bone formation and resorption.	OPG	RANKL, osteoclastogenesis, Th2 cells produced in the presence of IL-4 prevent bone loss.	[179,219,220,221,245]
IL-6	IL-12	Inhibits osteoblast differentiation.	Directly and indirectly supports osteoclast development while restricting the differentiation of osteoclast progenitors into osteoclasts.	IL-6 plays a critical role in the interaction between osteocytes and bone metabolism. IL-6, secreted by osteocytes in response to mechanical stress, regulates bone remodeling, osteoclastogenesis, and osteoblast activity. It has been shown that IL-6 enhances osteocyte-mediated osteoclastic differentiation via the JAK2/STAT3 pathway and is important in the modulation of bone mass.	Osteogenic capacity, bone resorption, osteoclast differentiation and activation, osteoclastogenesis, rheumatoid arthritis, cancer metastasis, atherosclerosis, and type 2 diabetes.	Osteoblast differentiation, bone trabecular volume.	[129,154,243,247,248,249]
IL-8	IL-8	-	Promotes RANKL-induced osteoclastogenesis.	Osteocytes, embedded within the bone matrix, play a crucial role in bone remodeling and are sensitive to various signaling molecules, particularly inflammatory cytokines like IL-8. These cells communicate with neighboring osteoblasts and osteoclasts through canaliculi, thereby regulating bone homeostasis. It is possible that IL-8 may affect osteocyte function and potentially contribute to bone remodeling processes.	Bone resorption, neutrophil RANKL expression.	Bone density	[11,129,184,250,251]
IL-10	IL-10	Suppresses osteogenic activity in the bone marrow.	Inhibits the differentiation of osteoclast progenitors into osteoclast precursors and suppresses RANK-induced osteoclast formation.	IL-10 plays a significant role in bone metabolism and regulates osteoclastogenesis. IL-10 inhibits bone resorption by upregulating the expression of OPG while downregulating the expression of RANKL and colony-stimulating factor-1 (CSF-1). Additionally, IL-10, in conjunction with IL-4, promotes the osteogenic differentiation of osteoblasts and induces an anti-inflammatory phenotype in macrophages. This indicates that IL-10 both inhibits bone resorption and supports bone formation.	Bone formation, bone fragility.	Inhibits osteoclast differentiation, reduces bone resorption, bone loss, reduced osteoclast production, decreased bone mass. It suppresses osteogenic differentiation by inhibiting bone mineralization and synthesis of bone proteins.	[211,245,252,253]
IL-13	Th2	-	Inhibits osteoclast formation and bone resorption.	Osteocytes play a crucial role in bone remodeling and are sensitive to cytokines. IL-13, known for its anti-inflammatory properties, may affect osteocyte function and bone metabolism. Although the specific effects of IL-13 on osteocytes are not well-detailed, it is possible that IL-13, like IL-10 and IL-4, modulates osteocyte activity and bone homeostasis.	Bone formation, bone resorption control, and bone mass preservation, bone tissue strength.	Bone loss	[217,224,225]
IL-17	IL-17	Enhances the expression of cytokines such as TNF-α, IL-6, and RANKL that promote osteoclasts in osteoblasts. Supports osteoblast differentiation while preventing osteoblast calcification.	Initiates osteoclast formation. At low concentrations, it enhances autophagy and osteoclast formation in osteoclast precursors, while at high concentrations, it inhibits the differentiation of osteoclast precursors into osteoclasts.	IL-17, a proinflammatory cytokine, has significant effects on osteocytes. IL-17 promotes bone resorption by regulating RANKL production in osteocytes. Additionally, IL-17 receptor signaling mediates PTH-induced bone loss and increases osteocytic RANKL production. IL-17 also promotes bone resorption and fluid shear stress regulation through osteocyte-specific signaling pathways.	Bone loss, RANKL, pro-osteoclastogenic cytokine.	Bone resorption	[11,147,174,177,178,179,180,254,255,256]
IL-18	IL-1	-	Inhibits TNF-α-induced osteoclastogenesis through apoptosis via Fas/FasL and NO in myeloid cells. Indirectly inhibits osteoclast formation through IFN-γ and GM-CSF.	Since osteocytes are sensitive to various signaling molecules, it is possible that IL-18 could affect osteocyte function and bone metabolism. Further research is needed to understand the specific effects of IL-18 on osteocytes.	In the presence of TNF-α, IL-12 and IL-18 synergistically increase NO production. An increase in IL-18 level causes a decrease in IL-18BP level. TNF-α, IL-1β, and IL-6 increase osteoclast differentiation and activation.	Osteoclast differentiation and activation.	[186,187,188,189,190,191]
IL-23	IL-12	-	Participates in T-cell-mediated osteoclast formation, regulates osteoclast differentiation, and indirectly inhibits osteoclast formation.	It is possible that IL-23 could affect osteocyte function and bone metabolism. Given its role in inflammation and immune responses, IL-23 may regulate bone remodeling and cell communication. Further research is needed to understand the specific effects of IL-23 on osteocytes.	It transforms CD4(+) cells into Th17 cells, thus increasing the production of IL-17 by Th17 cells. Osteoclast differentiation and activation.	Osteoblast apoptosis.	[177,178,199,200,201,202]
IL-33	IL-1	Stimulates osteoblast function, promotes matrix mineral deposition, and reduces sclerostin mRNA.	Terminates the osteoclast formation initiated by RANKL and suppresses the gene expression associated with osteoblasts. It induces apoptosis in osteoclasts while preventing osteoclast formation and bone resorption induced by TNF.	IL-33 has been shown to induce IL-6 expression and regulate osteocyte function by interacting with ST2L receptors on the plasma membrane and activating specific signaling pathways. These findings suggest that IL-33 may play a significant role in regulating osteocyte function and bone metabolism, highlighting a potential connection between IL-33 and osteocytes.	IL-33 increases during inflammation, increasing osteoblast activity.	Inhibiting osteoclast formation.	[11,13,205,206,207,208,257]
IL-35	IL-12	Promote the differentiation of mesenchymal stem cells into osteoblasts.	Prevent TNF-induced osteoclast formation and promote apoptosis, support the formation of functional osteoclasts, and increase the expression of osteoclast differentiation factors.	The connection between IL-35 and osteocytes has not been directly addressed in the literature. However, based on the effects of cytokines on bone cells, it can be hypothesized that IL-35 may influence osteocyte function and bone metabolism. Further research into the role of IL-35 in bone remodeling, osteoclastogenesis, and osteoblast activity could provide insights into this connection.	JAK1/STAT1, β-catenin and Axin2, bone formation and resorption.	Osteoclast formation, TNF-induced osteoclastogenesis and bone resorption, NF-κB, MAPK, NFATc1, c-Fos, and TRAP, apoptosis and adipogenic differentiation, adipogenesis.	[11,228,230,231,232,233,258]

### 2.2. GFs

GFs, also known as cytokines, are endogenous proteins that influence a wide range of cells by binding to cell surface receptors and directing their activation [259]. During bone healing, cytokines and cells cooperate to initiate bone repair. These polypeptides act locally as modulators of cellular activities [260]. Their effects can be autocrine (affecting the cell of origin or cells of the same phenotype), paracrine (affecting neighboring cells of a different phenotype), or endocrine (affecting cells located in distant anatomical regions). A single GF can exert effects on multiple cell types and bind to target receptors, inducing intracellular signaling that reaches the nucleus and determines the biological response [261]. 

The main growth factors affecting the skeleton include IGFs, TGFs, FGFs, epidermal growth factor (EGF), hepatocyte growth factor (HGF), vascular endothelial growth factor (VEGF), platelet-derived growth factor (PDGF), and nerve growth factor (NGF). Both the GFs embedded within the bone matrix and those secreted by bone and reactive cells contribute to the bone repair process.

#### 2.2.1. IGFs

IGF-1 is locally produced by osteoblasts, which are regulated by GH and other factors such as PTH and PGE2, which increase IGF-1 mRNA levels [262,263]. Intermittent administration of PTH maximizes the anabolic effects of IGF-1, whereas continuous infusion reduces IGF-1 expression [264]. IGF-1 increases type I collagen synthesis, ALP activity, and osteocalcin production in osteoblasts while reducing apoptosis and supporting osteoblastogenesis through activating the Wnt signaling pathway [265,266,267]. In elderly and osteoporotic individuals, bone formation decreases, and reduced levels of GH and IGF-1 contribute to bone loss [268,269]. 

IGF-1 and IGF-2 function as autocrine regulators of osteoblastic cell activity. However, the correlations between IGF-2 levels and BMD are not definitive [59]. Several studies have reported no relationship between IGF-2 and BMD [270,271,272,273]. Osteoclasts express IGF-1 receptors, and IGF-1 can directly affect these cells. In vitro studies have shown that IGF-1 induces the synthesis of RANKL and, subsequently, osteoclastogenesis. Thus, understanding the effects of GH and IGF-1 on bone cells is essential for developing treatments for osteoporosis [266,274,275].

#### 2.2.2. TGFs

TGFs, known as TGF-alpha (TGF-α) and TGF-β, are peptide growth factors belonging to the EGF and cytokine families, encoded by the TGFA and TGFB genes, respectively. They play crucial roles in cellular proliferation, differentiation, homeostasis, growth, and apoptosis [276,277]. TGF-α, produced by osteoclasts, controls the osteoblastogenesis of stromal cells [278]. Their involvement in immune regulation, embryonic development, tissue repair, wound healing, and tumor progression underscores their indispensability in biological systems. These factors differ in structure and function, binding to various receptors and following distinct signaling pathways [276,277]. 

TGF-β plays a crucial role in bone homeostasis in mammals, with three isoforms: TGF-β1, TGF-β2, and TGF-β3 [279]. TGF-β1, secreted by osteoblasts and osteoclasts, is involved in bone formation, hematopoietic cell formation, and mineral storage [280]. During the early stages of bone formation, TGF-β1 promotes the proliferation and migration of progenitor cells, while it inhibits osteoblast differentiation in later stages [281,282]. The effect of TGF-β1 on osteoclasts is concentration-dependent; low levels promote the migration and maturation of osteoclast precursors to resorption sites, whereas high levels inhibit this process. However, alterations in TGF-β signaling lead to various skeletal disorders, decreased bone mass, and poor bone quality [283,284]. 

Studies have revealed that in TGF-β1-deficient mice, tibia length is reduced, and bone mineral content decreases. TGF-β3 deficiency results in palate shelf fusion defects. Elevated TGF-β1 concentrations enhance osteoclast activity, leading to osteoarthritis, while excessive osteoblastic TGF-β2 expression causes increased bone remodeling and progressive bone loss [285]. 

BMP and TGF-β signaling also play significant roles in osteoclast differentiation. BMP signaling supports osteoclast formation by increasing the RANKL/OPG ratio and promoting the expression or activity of osteoclastic transcription factors. Conversely, TGF-β regulates osteoclast formation in a dose- and stage-dependent manner; low doses induce the migration of osteoclast precursors to resorption pits, whereas high doses inhibit this process [286,287,288].

#### 2.2.3. FGFs

The FGF family plays critical roles in tissue homeostasis, repair, regeneration, angiogenesis, and bone metabolism [14,289]. This large family of proteins is crucial for bone health and metabolism, and the effects of various FGF members on bone have been extensively studied [290,291].

##### FGF-2

FGF-2 plays a significant role in regulating bone regeneration and cartilage differentiation. It is expressed in osteoblasts, stored in the ECM, and acts as a fundamental regulator of osteoblast function. Disruption of FGF-2 can lead to reduced osteoblast replication and impaired new bone formation [292,293]. Additionally, FGF-2 facilitates bone anabolism mediated by PTH through the Wnt/β-catenin signaling pathway [294]. This pathway is vital for bone growth, development, and fracture healing. The therapeutic potential of FGF-2 is promising, as it may support osteogenesis, fracture healing, and the regulation of bone calcium–phosphate balance [295].

##### FGF-19

FGF-19 is secreted by ileal epithelial cells and regulates hepatic glycolipid and bile acid metabolism [296]. It forms a complex with FGFR4 and β-klotho. In postmenopausal osteoporosis (PMO) patients, FGF-19 levels are found to be lower than in healthy women and positively correlate with BMD [297]. FGF-19 is suggested to improve bone loss by promoting osteoblast differentiation and inhibiting osteoclastogenesis. Additionally, it contributes to bone metabolism by regulating bile acid metabolism [293,297,298].

##### FGF-21

FGF-21 is highly expressed in the liver, adipose tissue, and muscles. It plays a significant role in metabolic processes, increasing insulin sensitivity in diabetic mice but showing no effect on bone mass or biomarkers in obese mice induced by a high-fat diet [296,299]. Studies on the relationship between FGF-21 and BMD have yielded inconsistent results in humans. Experimental studies have shown that exogenous FGF-21 increases bone resorption, decreases bone formation, and reduces BMD [300,301]. Some studies suggest a negative correlation between serum FGF-21 and BMD, while others find no significant relationship [302,303]. The role of FGF-21 involves the regulation of glucose and lipid metabolism and the secretion of factors related to muscle tissue function, indirectly affecting bone mass. However, further research is needed to determine the specific impact of FGF-21 on BMD and whether it directly affects bone tissue in specific pathological conditions [304,305].

##### FGF-23

FGF-23 is primarily produced by osteocytes and regulates phosphate and 1,25-dihydroxyvitamin D3 [306]. It reduces phosphate reabsorption in the kidneys and inhibits the expression of 1-hydroxylase, thus interfering with Ca homeostasis [307]. High levels of FGF-23 are associated with hypophosphatemia, leading to rickets in children and osteomalacia in adults [291]. The autocrine/paracrine effects of FGF-23 on bone mineralization are independent of Klotho and regulate bone mineralization by inhibiting tissue non-specific alkaline phosphatase (TNAP) [308]. FGF-23 is associated with increased osteoblastic activity and ALP bone nodules. It also regulates bone mineralization by controlling osteopontin expression. Supra-physiological concentrations of FGF-23 inhibit TNAP activity through the FGFR3 extracellular signal-regulated kinase (ERK) pathway, increasing extracellular PPi and reducing inorganic phosphate, thereby decreasing osteopontin expression. The effects of FGF-23 on osteoblast differentiation and activity can vary under physiological and supra-physiological conditions [308,309].

The relationship between FGF-23 and BMD has yielded mixed results in various observational studies. Coulson et al. reported no significant association between FGF-23 and whole-body BMD [310]. FGF-23 levels were similar between young (18–30 years) and older (69–81 years) participants. A prospective study in the elderly population (70–79 years) in the USA also found no significant relationship between FGF-23 levels and annual percentage changes in total hip BMD after adjusting for demographics, BMI, and estimated glomerular filtration rate (eGFR) [311]. In contrast, Isakova [311] and Jovanovich [312] also reported no significant association between FGF-23 levels and fracture risk in the elderly population, regardless of gender.

Among Caucasian and African-American premenopausal women, a negligible association between FGF-23 and BMD at the spine and femoral neck was reported [313]. A study among Chinese postmenopausal women also found no significant correlation between FGF-23 and lumbar BMD [314]. In contrast, Celik et al. found that postmenopausal women with osteoporosis had significantly higher FGF-23 levels compared to those with osteopenia and healthy controls [315]. Lane et al. found no significant relationship between FGF-23 levels and the incidence of spine, non-spine, major osteoporotic fractures, or vertebral fractures among elderly Swedish men [316].

In contrast, some studies have reported a significant association between FGF-23 levels and the risk of osteoporotic fractures. Mirza et al. found a significant association between higher FGF-23 levels and an increased risk of vertebral fractures among 2868 elderly Swedish men, but no association with non-vertebral fractures [317]. The fracture risk was highest when FGF-23 levels exceeded 55.7 pg/mL. Similarly, a study in the Japanese population with early-stage chronic kidney disease (CKD) reported an optimal cutoff point of 56.8 pg/mL for FGF-23 levels in predicting vertebral fractures [316]. 

#### 2.2.4. EGF

The EGF family comprises structurally and functionally similar proteins. In recent years, members of the EGF family have been identified as playing significant roles in bone biology [318,319]. EGF enhances the proliferation and migration of bone marrow stromal cells (BMSCs), which can lead to the formation of osteoblasts, chondrocytes, and adipocytes, thus serving as a source for mesenchymal tissue regeneration [320,321].

Stimulation of BMSCs with EGFR ligands increases the production of growth and differentiation factors and cytokines, such as VEGF, angiopoietin-2, PDGF-BB, granulocyte-colony stimulating factor (GCSF), HGF, IL-6, and IL-8. Activation of EGFR in BMSCs and osteoblasts triggers similar signaling pathways to those in other target tissues [320,321,322].

Proinflammatory cytokines, such as IL-6 and IL-8, are direct targets of EGF and participate in the inflammatory phase of fracture healing/bone regeneration through non-canonical Wnt signaling via Wnt-5a and ROR2. However, recent studies have also shown that EGFR signaling maintains osteoblasts in an undifferentiated state, inhibits the expression of key osteogenic differentiation markers, and is essential for the anabolic effects of intermittent PTH treatment [323,324,325,326].

#### 2.2.5. HGF

HGF, identified in 1984, is a cytokine that promotes the proliferation of hepatocytes [327]. Research has demonstrated that HGF is a multifunctional GF secreted by various cell types [328,329]. Also known as scatter factor, HGF is a pleiotropic molecule that regulates cell proliferation, motility, morphogenesis, and angiogenesis. Additionally, HGF has been shown to play significant roles in organogenesis, tissue repair, neuronal induction, and bone remodeling [330,331].

In terms of its effects on bone, HGF exhibits dual actions by expressing the c-MET receptor in both osteoblasts and osteoclasts, and is synthesized by osteoclasts [332]. HGF promotes the osteoblastic differentiation of mesenchymal stem cells, the proliferation of osteoblasts, and osteogenesis, contributing to improved fracture healing in animal models [333]. Moreover, HGF has been reported to stimulate migration, cell division, and morphological changes in osteoclasts [334]. 

According to a study by Chen et al., HGF, when used in combination with the osteogenic differentiation activator 1,25-dihydroxyvitamin D3 (1,25OHD), can induce osteogenic differentiation of human MSCs (hMSCs) by upregulating the expression of the 1,25OHD receptor, Vitamin D Receptor (VDR) [335].

#### 2.2.6. VEGF

VEGF belongs to a homodimeric protein family consisting of six members: VEGF-A, VEGF-B, VEGF-C, VEGF-D, VEGF-E, and Placental Growth Factor (PlGF) [336,337]. VEGF plays a critical role in osteogenesis by promoting angiogenesis and vascularization. Additionally, it regulates bone remodeling by stimulating osteoblast differentiation and survival [338,339]. Various hormones affect skeletal homeostasis by regulating local VEGF production, though their impact on circulating VEGF levels remains unclear. Notably, estrogen has been shown to regulate VEGF gene transcription [340,341].

In bone development and repair, several growth factors regulate VEGF expression. These factors include (TGF-β, BMPs), IGF, and FGF. Moreover, inflammatory factors and mechanical stress also influence VEGF expression. VEGF accumulates in hematomas and initiates bone repair during inflammatory responses. Osteoblasts play a significant role in VEGF secretion and neutrophil chemotaxis [342,343,344,345,346].

Osteoclasts are critically involved in bone resorption and differentiation, with VEGF enhancing osteoclast survival and bone resorption activity [347,348]. Functional VEGF receptors (VEGFRs) have been identified in primary osteoblasts, indicating that VEGF directly affects osteoblast survival, chemotactic migration, and activity [349,350]. Studies have shown that osteoblastic cells produce VEGF, which is involved in the paracrine regulation of blood vessel formation in bone tissue and the autocrine regulation of osteogenesis [351,352].

PDGF is another important factor for bone regeneration [353]. VEGF can inhibit PDGFR activation, leading to the formation of immature blood vessels [354]. The inhibitory effects of VEGF on osteoblasts should be considered in bone repair and regeneration strategies [355,356].

#### 2.2.7. PDGF

PDGFs are GFs composed of polypeptide dimers linked by disulfide bonds, and are synthesized by various cell types including platelets, macrophages, osteoblasts, and fibroblasts [357]. PDGF acts as a crucial mitogen for osteoblasts, fibroblasts, smooth muscle cells, and glial cells [358]. It supports organogenesis, skeletal development, angiogenesis, and wound healing. In vitro studies have shown that PDGF is expressed in osteoblasts and osteoclasts and increases the production of osteoprotegerin [359]. The presence of PDGF receptors on osteoblasts and osteoclasts indicates its mitogenic effect on these cells [359,360,361].

PDGFs exist in five dimeric forms: PDGF-AA, PDGF-BB, PDGF-AB, PDGF-CC, and PDGF-DD. PDGF-BB regulates bone formation and plays a significant role in fracture healing [362,363]. PDGF-BB produced by osteoclasts enhances angiogenesis, and its deficiency leads to reduced bone mass [364]. PDGF-BB promotes the infiltration of mesenchymal and angiogenic progenitor cells and regulates chondrogenic and osteogenic responses. However, the direct effect of PDGF-BB on osteoclast formation and precursor chemotaxis remains unclear [362,365].

Cytokines such as RANKL, M-CSF, and TNF-α affect osteoclast formation by regulating signaling molecules like NF-κB, NFATc1, B cell lymphoma (BCL) 2, dendritic cell specific transmembrane protein (DC-STAMP), and c-Fos [366,367,368]. The signaling pathways ERK1/2, JAK2/STAT3, and Akt positively regulate osteoclast formation and precursor cell chemotaxis. The effects of PDGF-BB on osteoclast precursor cells are still under investigation [369,370].

#### 2.2.8. NGF

NGF is one of the four neurotrophic factors. NGF is essential for the maintenance, differentiation, and growth of peripheral sympathetic and sensory neurons as well as basal forebrain cholinergic neurons. Its neuronal origin and function are characterized by the modulation of nociception through changes in receptor sensitivity, gene expression, and local neuronal sprouting. Additionally, NGF affects immune-hematopoietic cells (T and B lymphocytes, monocytes/macrophages, eosinophils, basophils, neutrophils), parenchymal cells (adipocytes, pancreatic beta cells, thyrocytes, keratinocytes, osteoblasts, mesenchymal cells), and structural cells (fibroblasts, vascular stromal cells) [371,372]. 

The postmenopausal period represents immune and metabolic changes due to estrogen deficiency [373]. ERs are expressed in immune cells (macrophages, T and B lymphocytes, dendritic cells, eosinophils, mast cells). The protective role of estrogen in energy, glucose, and hematopoietic homeostasis gradually diminishes during the postmenopausal period, leading to life-threatening conditions such as osteoporosis, cardiovascular diseases, tumors, and immune and hematopoietic disorders [374].

Estrogen deficiency leads to alterations in vascular structure and endothelial function, promoting the release of various mediators [IL-6, Monocyte Chemoattractant Protein 1 (MCP-1), RANTES (regulated upon activation, which normal T cell expressed and presumably secreted) chemokine, VEGF]. The regulation of VEGF is associated with the hypertrophy of vascular smooth muscle cells [375].

A study by Molnar demonstrated that increased NGF levels are associated with escalated MCP-1 levels along with postmenopausal osteoporosis. Dual-energy X-ray absorptiometry (DXA) measurement of patients revealed that an increased NGF level was associated with the osteoporosis of the lumbar spine but not of the total forearm and femoral neck [376]. 

## 3. Conclusions and Future Perspective

Osteoporosis is a multifaceted disease influenced by a variety of factors, including GFs and their effects, hormonal changes, cytokine activity, genetic predisposition, and lifestyle choices. The intricate balance between bone resorption and formation is regulated by a network of hormones and cytokines, which play crucial roles in maintaining bone health and density. This review has highlighted the significant impact of estrogen, testosterone, PTH, and calcitonin on bone metabolism, emphasizing their roles in the pathophysiology of osteoporosis. Additionally, the interplay of pro-inflammatory and anti-inflammatory cytokines complicates osteoporosis management because it affects the balance of bone breakdown and formation, making it difficult to predict and control the disease.

Current diagnostic methods can benefit from advancements in technology to enhance early detection and improve treatment outcomes. Emerging diagnostic tools and biomarkers, such as osteocalcin and osteopontin, are promising for more precise and individualized approaches for osteoporosis management. Understanding hormonal and cytokine influences on bone health is essential for developing targeted therapies that address the underlying mechanisms of bone loss.

Future research should focus on exploring new therapeutic strategies that modulate hormonal and cytokine activity, as well as investigating the potential of novel biomarkers for early diagnosis. By integrating these advancements into clinical practice, it is possible to improve the quality of life of individuals affected by osteoporosis and reduce the global burden of this prevalent disease.
